# Extended-spectrum beta-lactamases from French veal calves: WGS-based data will help build targeted AMR mitigation strategies

**DOI:** 10.1128/spectrum.00659-26

**Published:** 2026-06-22

**Authors:** Marisa Haenni, Véronique Métayer, Antoine Drapeau, David le Goïc, Jean-Yves Madec

**Affiliations:** 1 ANSES – Université de Lyon – Unité Antibiorésistance et Virulence Bactériennes, Lyon, France; 2 Clinique AmiVet, Janzé, France; 3 Clinique Vitelliance, Réseau Cristal, La Guerche de Bretagne, France; Institut National de Santé Publique du Québec, Sainte-Anne-de-Bellevue, Québec, Canada

**Keywords:** veal calves, French dairy farms, *E. coli*, ESBL/AmpC

## Abstract

Antimicrobial resistance (AMR) is an important One Health issue affecting the three human, animal, and environmental sectors. The occurrence of *Escherichia coli* resistant to extended-spectrum cephalosporins (ESC-R) is considered a relevant marker for the estimation of the AMR burden. In the dairy industry, veal calves present the highest proportions of ESC-R compared to adults. Since little is known in France about the AMR dynamics from birth to the fattening farm, our observational study determined the proportion of ESC-R *E. coli* using selective media, and characterized by whole-genome sequencing. Bacterial isolates identified at the farm level and at the sorting center, before being sent to the fattening farms. The proportion of ESC-R-positive calves coming from farms in which waste milk was given to calves (30.3%) was higher than in the ones where it was discarded (13.1%). Overall, 12.5% of the veal calves were ESC-R carriers in their farm of origin, and 6.2% of the additional animals became carriers during their transport to the sorting center. Over the 176 ESC-R *E. coli* isolates sequenced, 55.1% (*n* = 97) presented an extended-spectrum beta-lactamase phenotype conferred by a *bla*_CTX-M_ gene, while 44.9% (*n* = 79) presented an AmpC phenotype due to chromosomal mutations. A few specific clones (ST8095 and ST117) were identified as responsible for AMR dissemination across farms. Our results showed that AMR in dairy calves results from cumulative effects of all risk factors along the whole production chain, including feeding calves with antibiotic residues-containing waste milk, animal grouping, and insufficient biosecurity measures in dairy farms.

**IMPORTANCE:**

Despite the implementation of national action plans, the presence of *Escherichia coli* resistant to extended-spectrum cephalosporins (ESC-R), mainly due to the presence of extended-spectrum beta-lactamases or AmpCs, is still high in many veal-producing countries. In France, previous studies reported antimicrobial resistance (AMR) data during the fattening period and at the slaughterhouse, but data are still scarce on the AMR status of veal calves on their farm of origin. This study aimed at determining the proportion of ESC-R *E. coli* and characterizing bacterial isolates identified at the farm level and at the sorting center, just before being sent to the fattening farms. This is of utmost importance since mathematical models showed that minimizing AMR prevalence upon arrival in the fattening farm is an important factor to mitigate ESC-R carriage during the fattening process. In addition, we are providing important WGS data from the veal calf sector, which can be compared to already-existing data.

## INTRODUCTION

Antimicrobial resistance (AMR) is a public health issue, and addressing this problem in livestock has become a priority over the last few decades. According to the World Health Organization (WHO) bacterial priority pathogens list revised in 2024 (1), Enterobacterales resistant to extended-spectrum cephalosporins (ESC-R) remain among the three top major concerns with carbapenem-resistant (CP-R) Enterobacterales and *Acinetobacter baumannii*, causing treatment failures and increased health costs (2). While CP-R is still mostly restricted to humans, ESC-R Enterobacterales have been widely disseminated in all human, animal, and environmental sectors, so that ESC-R *E. coli* are commonly admitted as a global marker of the AMR burden. ESC-R resistance is mostly conferred by the production of extended-spectrum beta-lactamases (ESBLs), but also by the overexpression of the *ampC* gene, either due to chromosomal mutations in the promoter region of the gene or to the acquisition of plasmidic *ampC* genes such as *bla*_CMY-2_ or *bla*_DHA-1_.

In the dairy industry, several studies showed that veal calves represent the highest proportion of AMR compared to adults (3–6). The microbiota of calves evolves dramatically in the first weeks of life due to diet transition, with, for example, an important relative abundance of *Escherichia*, *Streptococcus*, and *Enterococcus* genera during early days, which then decreases over time (7, 8). The microbiota of pre-weaned animals is especially heterogeneous and prone to acquire resistant bacteria (6, 8). These resistant bacteria can originate from the farm environment but can also be selected from the gut when calves are fed with the colostrum (9) or waste milk of treated cows containing residues of antibiotics, a widespread practice that the European Food Safety Authority (EFSA) advised against (10). In the French intensive system, calves are collected at 3–5 weeks of age from their farm of origin, batched in a sorting center, and then placed in dedicated farms for the fattening period (5–6 months). Consequently, several external factors such as stress, disease, and subsequent antibiotic treatments can influence their microbiome and resistome.

The proportion of ESC-R *E. coli* in veal calves is often reported as high: 48% in the Czech Republic (11), 22.5% in Germany (12), 21.2% in Austria (13), 19.3% in the Netherlands (6), or 17.1% in Italy (14). In France, a study in 2014 showed that the prevalence of ESC-R *E. coli* was high (67.7%) when calves arrived at the fattening farm and then decreased to 20.4% just before being slaughtered (15). This decrease in ESC-R *E. coli* was usually achieved within a few weeks after entering the fattening farm, depending on the general health status of the animals and the antibiotic treatments throughout the fattening process (16). While AMR data and trends are available for the fattening period, information on AMR beforehand—from birth to the fattening farm—is lacking. Indeed, during these 3–5 weeks, several factors might contribute to AMR selection and spread within and between calves, such as exposure to antibiotics or antibiotic residues, animal grouping on farm and/or during transportation, or contacts with professionals (veterinarians, inseminators, or other breeders).

This study addressed this knowledge gap by determining the proportion of ESC-R *E. coli* and characterizing bacterial isolates identified at the farm level and when calves arrived at the sorting center, just before being sent to the fattening farms. We assessed whether AMR was more likely acquired on birth farms (due to antibiotic therapy or feeding calves waste milk, for instance), and/or whether other factors (such as transport or grouping of animals) might play a role, so that professional organizations would be able to take appropriate measures to decrease the AMR burden in the calf sector.

## MATERIALS AND METHODS

### Sampling scheme

**Fig 1:**
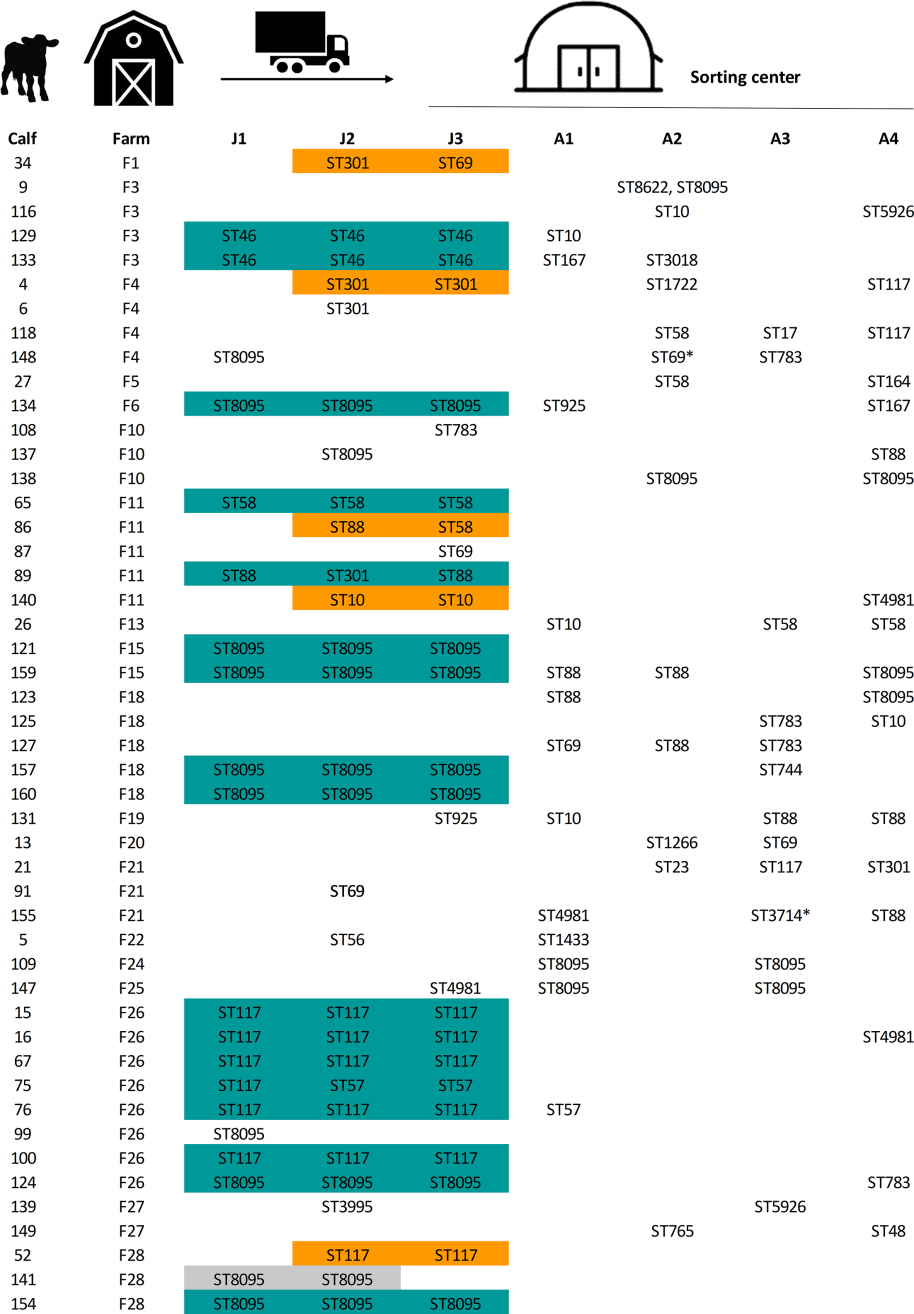
Schematic representation of all ESC-R isolates sequenced. The first “calf” column indicates the calf identifier, the second “farm” column indicates the code of the farm of origin, and the J1, J2, J3, and A1-A4 columns contain the ST of each sequenced isolate. Calves that were positive at J1, J2, and J3, STs are highlighted in dark green. When a calf was positive at J2 and J3, STs are highlighted in light orange. For the only calf that was positive at J1 and J2, STs are highlighted in gray.

One hundred and sixty veal calves, called index calves, were followed from their birth farm to the sorting center. These 160 veal calves, which originated from 31 different farms (Table S2), were sampled three times: (i) in the farm where they were born (J1), (ii) at their entry into the sorting center (J2), and (iii) at their departure from the sorting center (J3) to the fattening farm, representing 480 samples (Fig. 1). In the sorting center, these 160 calves were mixed with animals from other farms and gathered in pens, grouping 10 individuals. At the departure from the sorting center, for each index calf, four additional calves (A1–A4) sharing the same pen were also sampled, representing 640 samples (Table S1). For these A1–A4 calves, no information was available on their birth farm. All calves were sampled by the same veterinarian. The sampling period spanned from September 2019 to September 2020.

For each index calf (*n* = 160), a questionnaire was filled by the farmer, indicating (i) the type of farm (organic/conventional), (ii) potential health issues on farm (i.e., frequent diarrhea), (iii) feeding calves waste milk (withdrawn for antibiotic use or not) or not, and (iv) whether the calf sampled had received antibiotics (Table S2). The questionnaire was validated by the veterinarian who sampled all animals.

### Bacterial isolation and identification

Rectal swabs from each calf were taken using eSwabs (Biomerieux), placed in portable coolers with ice packs, and shipped to the Anses laboratory in Lyon, France, where they were processed immediately. Upon their arrival, swabs were plated on selective ChromID ESBL agar (Biomerieux) for the detection of ESC-resistant *E. coli,* as well as on MacConkey agar as a control of growth (data not shown). One presumptive *E. coli* colony was arbitrarily selected from the ESBL agar plate and identified using colony morphology and a species-specific PCR.

### Antimicrobial susceptibility testing

Susceptibility testing was performed using disc diffusion on Mueller-Hinton agar (Bio-Rad, Marne-la-Coquette, France), according to the guidelines and clinical breakpoints (human and veterinary) of the Antibiogram Committee of the French Society for Microbiology https://www.sfm-microbiologie.org/2023/06/15/casfm-veterinaire-2023/ for veterinary-related antibiotics and https://www.sfm-microbiologie.org/wp-content/uploads/2023/06/CASFM2023_V1.0.pdf for human-related antibiotics. Antibiotics (Mast Diagnostics) of human and/or veterinary interest were tested (Table S1). *E. coli* ATCC 25922 was included for quality control both for disc diffusion and broth micro-dilution. Minimum inhibitory concentrations (MICs) for colistin were determined by broth microdilution, according to the EUCAST recommendations. All isolates presenting with resistance to three or more antibiotic families were considered MDR.

### Illumina short-read sequencing and data analyses

DNA was extracted using the NucleoSpin Microbial DNA extraction kit (Macherey-Nagel, Hoerdt, France) according to the manufacturer’s instructions. Library preparation (Nextera XT technology) and sequencing (NovaSeq-6000 instrument) were outsourced (Eurofins, Germany). Raw sequencing reads were processed using FastQC v0.12.1 for quality control and Trimmomatic v0.39 to remove low-quality reads. *De novo* assembly was performed using Shovill v1.0.4, and the quality of assemblies was assessed using QUAST v5.0.2 (Table S3). Identification was performed using Kraken (https://github.com/DerrickWood/kraken). Sequence types (STs), resistance genes, and virulence factors were determined using the CGE online tools (http://www.genomicepidemiology.org/) MLST v2.0, ResFinder v4.1, and VirulenceFinder 2.0.3, while replicon content and plasmid formula were identified using PlasmidFinder 2.0.1 and pMLST 2.0.

### Phylogenetic analysis

The cgMLST-based phylogeny of all sequenced isolates was determined through the pyMLST pipeline v2.0.1 (https://github.com/bvalot/pyMLST) using the scheme available on the https://www.cgmlst.org/ncs, which comprised 2,513 alleles for *E. coli*. The cut-off for highly related strains was <10 allelic differences. Distance matrix between isolates was performed using snp-dists v0.8.2 (Table S4).

SNP-based phylogenies were individually performed on all genomes belonging to STs that were identified in more than 10 isolates. An annotation was generated using Bakta v1.9.3, and the pangenome was constructed using the PPanGGOLiN pipeline v2.1.0. SNP distances (Table S4) were calculated using snp-dists v0.8.2.

All resulting trees were visualized using iTol v7 (https://itol.embl.de/).

## RESULTS

### Proportion of ESC-R isolates on farm (J1), after transportation (J2), and at departure from the sorting center (J3)

Veal calves (*n* = 160) originated from 31 different farms, all located in the same French department (Ille-et-Vilaine), and 1–16 different veal calves were sampled per farm (Table S2). The number of calves sampled depended on the number of animals sent to the sorting center at the time of sampling, and thus also on the size of the farm. Seven farms (7/31, 22.6%) were organic (Table S2). In 13 farms (13/31, 41.9%), veal calves were fed with milk from cows treated with antibiotics (tetracycline/neomycin in nine farms, amoxicillin/clavulanic acid in two).

In 16 farms (16/31, 51.6%), at least one veal calf carried an ESC-R *E. coli* isolate. Over the 160 veal calves sampled on their farm of origin (index calves), 20 presented an ESC-R *E. coli* at J1 (20/160, 12.5%), 28 at J2 (17.5%), and 26 at J3 (16.3%). Among these calves, 17 carried an ESC-R isolate at J1 + J2 + J3, 1 at J1 + J2, and 5 at J2 + J3 (Fig. 1). Two veal calves were positive only at J1, five only at J2, and four only at J3.

Fourteen calves were sick (mostly diarrhea) on the day of sampling, of which only one (calf #52) carried an ESC-R-positive *E. coli*. This calf had not been treated with antibiotics and had been fed with milk withdrawn for a reason that was not related to antibiotic use (Table S2). The proportion of ESC-R-positive calves was similar in organic (19.4%, 6/31 calves raised on organic farms) and in conventional farms (21.7%, 28/129 calves raised on conventional farms) (Table S2). The proportion of ESC-R-positive calves coming from farms in which waste milk was given to calves (30.3%, 23 ESC-R-producers/76 calves raised in such farms) was higher than that in farms where waste milk was discarded (13.1%, 11/84).

Over the 640 veal calves sampled concomitantly to the J3 calves, 101 presented one ESC-R *E. coli* isolate (among which the calf 09-A2 displayed two different isolates). Consequently, 15.9% of the calves (J3 + A1–A4; 127/800) carried an ESC-R *E. coli* isolate at the departure from the sorting center.

### Resistance to ESC and non-beta-lactam antibiotics

No carbapenem-resistant isolate was identified. Among the 176 ESC-R *E. coli* isolates sequenced (74 from index calves and 102 from A1–A4 concomitant calves), 97 (97/176, 55.1%) presented an ESBL phenotype, while 79 (79/176, 44.9%) presented an AmpC phenotype (Table S5; Fig. 1). The ESBL phenotype was due to the presence of the *bla*_CTX-M-1_ (*n* = 54), *bla*_CTX-M-15_ (*n* = 20), *bla*_CTX-M-32_ (*n* = 5), *bla*_CTX-M-55_ (*n* = 2), *bla*_CTX-M-14_ (*n* = 11), and *bla*_CTX-M-27_ (*n* = 5) genes. The AmpC phenotype was systematically due to mutations in the promoter region of the *ampC* gene, which were either located on the −32 (−32T > A, *n* = 7) or −42 (−32C > T, *n* = 72) nucleotide. No acquired *ampC* gene was identified.

Numerous additional resistances were identified (Table 1), especially to streptomycin and kanamycin (84.7% and 85.2%), sulfonamides (94.9%), and tetracyclines (92.0%). These resistances were mostly due to the *aph(3*′*)-Ia* (*n* = 124), *aph (6)-Id* (*n* = 145), *sul2* (*n* = 149), and *tet*(A) (*n* = 122) genes (Table S5). No isolate was resistant to amikacin, while only 1.1% (*n* = 2) were resistant to colistin due to the presence of the *mcr-1.1* gene. Globally, *E. coli* isolates presenting an ESBL phenotype displayed more associated resistances than those displaying an AmpC phenotype (Table 1).

**TABLE 1:** Resistance phenotypes associated with *E. coli* isolates resistant to extended-spectrum cephalosporins

	ESBL phenotype (*n* = 97)		AmpC phenotype (*n* = 79)		Total (*n* = 176)	
	No.	%	No.	%	No.	%
Streptomycin	77	79.4	72	91.1	149	84.7
Kanamycin	79	81.4	71	89.9	150	85.2
Amikacin	0	0.0	0	0.0	0	0.0
Apramycine	25	25.8	1	1.3	26	14.8
Gentamicin	43	44.3	10	12.7	53	30.1
Tobramycin	43	44.3	11	13.9	54	30.7
Netilmicin	42	43.3	2	2.5	44	25.0
Chloramphenicol	34	35.1	15	19.0	49	27.8
Florfenicol	26	26.8	12	15.2	38	21.6
Tetracycline	89	91.8	73	92.4	162	92.0
Colistin^*a*^	2	2.1	0	0.0	2	1.1
Sulfonamides	94	96.9	73	92.4	167	94.9
Trimethoprim	45	46.4	21	26.6	66	37.5
Nalidixic acid	49	50.5	13	16.5	62	35.2
Enrofloxacin	16	16.5	0	0.0	16	9.1

^*a*^ As determined by broth micro-dilution.

### Genetic diversity of ESC-R isolates

**Fig 2:**
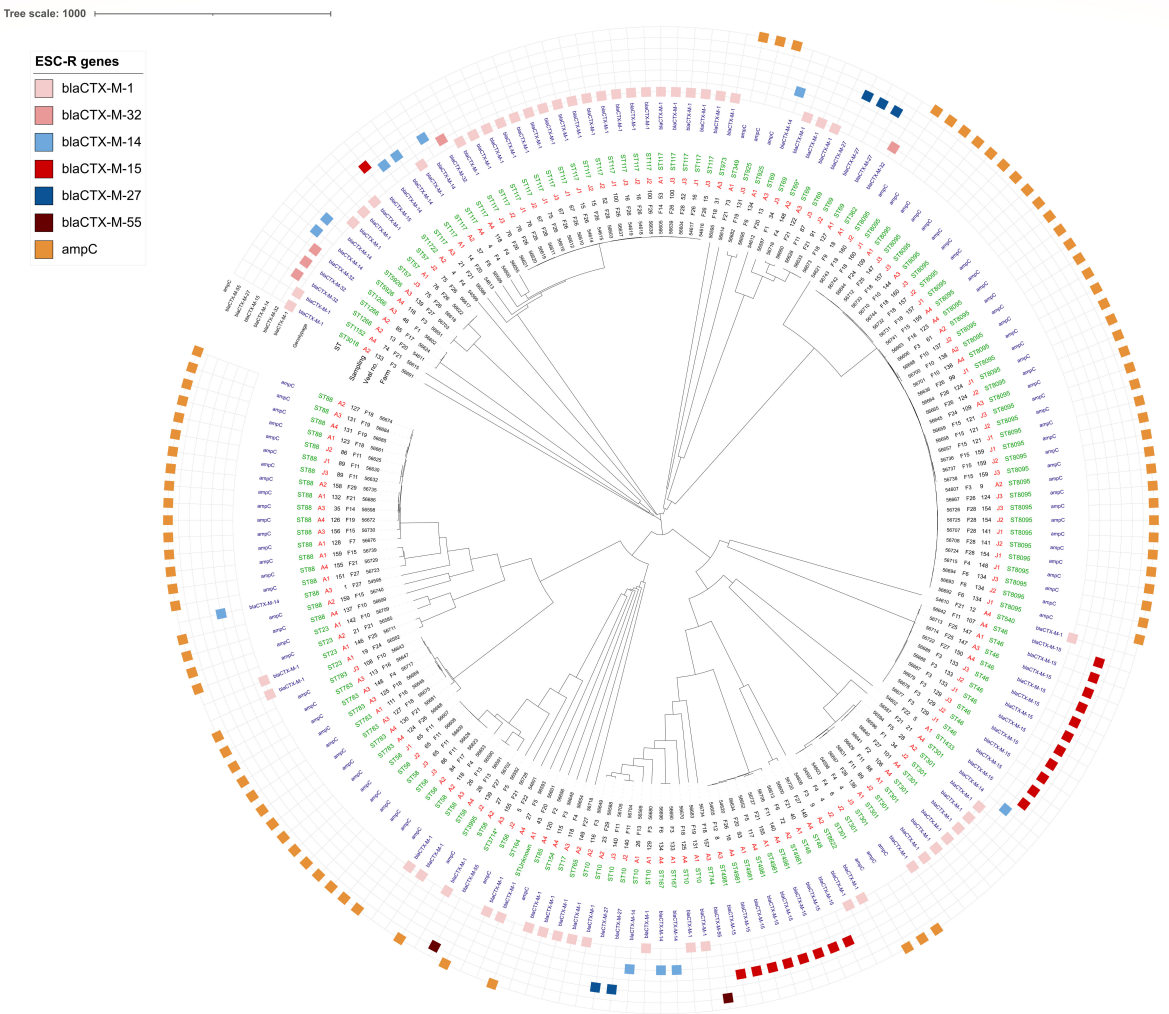
cgMLST-based phylogeny of all 176 sequenced *E. coli*. The presence of the *bla*_CTX-M_ genes and the over-expressed *ampC* gene is indicated by filled squares. Associated metadata (isolate identified, calf and farm number, corresponding ST) are indicated on the tree.

A high genetic diversity was observed, with 37 different STs identified over the 176 collected isolates (Fig. 1; Table S6). The most frequent STs were ST8095 (*n* = 36, 20.5%), ST117 (*n* = 24, 13.6%), ST88 (*n* = 19, 10.8%), ST301 (*n* = 11, 6.3%), and ST46 (*n* = 10, 5.7%), while 20 STs were singletons. The genetic diversity largely diverged between ESBL- and AmpC producers, with ST117 (*n* = 24) and ST46 (*n* = 10) being exclusively found among ESBL-producers, and ST8095 (*n* = 36) and ST88 (*n* = 18) being mostly exclusively identified (except one ESBL-producing ST88 isolate) among AmpC producers. Some STs were systematically associated with one specific resistance gene (or mutation) (Fig. 2), like ST8095 always presenting mutations in the promoter region of the *ampC* gene, or ST46 and ST4981 always displaying the *bla*_CTX-M-15_ gene. Conversely, most STs presented different resistance determinants, such as ST117 carrying either the *bla*_CTX-M-1_, *bla*_CTX-M-32_, or *bla*_CTX-M-15_ gene depending on the farm of origin. Several STs comprised isolates able to resist to ESC either due to either mutations in the promoter of the *ampC* gene or to the presence of a *bla*_CTX-M_ gene (ST23, ST58, ST88, and ST301) (Fig. 2).

### Clonality of ESC-R isolates

In cases when one index calf presented an ESBL- or AmpC-producing *E. coli* at J1, J2, and/or J3, isolates mostly belonged to the same ST and were clonally related (Fig. 2, Table S4). The only exceptions were for farm F11 (in which the calf carried an ST88 at J1 and J3, and an ST301 at J2), farm F26 (in which the calf carried an ST117 at J1 and an ST57 at J2 and J3), and farm F1 (in which the calf carried an ST301 at J2 and an ST69 at J3) (Fig. 1). When one ESC-R-positive calf (A1 to A4) shared the same pen as an ESC-R-positive index calf (J1–J3) in the sorting center, the *E. coli* isolate identified never belonged to the same ST. There was only one exception for calf #159, where calf A4 shared the same ST as the index calf at J1 to J3 (Fig. 1). The three J1, J2, and J3 isolates differed by a maximum of eight SNPs (Table S4), while the A4 isolate differed by 25 SNPs, most likely indicating that the A4 calf imported this *E. coli* from its farm of origin rather than acquired it from calf #159.

A long-term persistence of specific ESC-R clones was observed in a few farms. In farm F26, for example, a highly similar *bla*_CTX-M-1_-producing ST117 clone differing by 0–18 SNPs was observed in September 2019, and then in January and February 2020. This clone was also identified in farms F14 and F28, with isolates that only diverged from the F26 ones by a maximum of 18 SNPs (Table S4). Five other ST117 isolates were identified in this study, which clearly diverged (316–450 SNPs) and displayed different resistance genes (*bla*_CTX-M-14_, *bla*_CTX-M-15_, and *bla*_CTX-M-32_). These five clones were collected from calves A1–A4 that shared the same pen as the index calves at the sorting center. In farm F15, a highly similar ST8095 clone (4–17 SNPs) presenting mutations in the promoter of the *ampC* gene was identified in June and September 2020. In most cases, when several calves from a single farm presented ESC-R *E. coli* isolates, the identified clones belonged to the same ST and were clonally related, as for example in farms F3, F15, or F18. In a few other cases (farms F11 or F26), more than one ST was identified (Fig. 1).

## DISCUSSION

Concerns of AMR have been raised a long time ago in the veal sector, and high rates of ESC-R *E. coli* were regularly reported in Europe. Proportions identified in newborn calves on farm largely vary. To only cite a few studies, >90% of the calves were ESC-R-positive in one study in Germany (17), around 50% in the Czech Republic (11), and >25% in another study in Germany (12), in Austria (13), in the Netherlands (6), or in France (this study). This variability among studies reflects differences in the age of animals (with a clear decrease of ESC-R *E. coli* with age), farm structures (intensive vs organic, number and potential turnover of animals), and specific practices such as feeding calves with waste milk. Indeed, the presence of antibiotic residues in waste milk was shown to select resistant bacteria (18, 19), and the EFSA clearly advised against this practice in its 2017 report (20). In this study, the proportion of ESC-R *E. coli* was higher in farms reporting feeding calves with waste milk (30.3% versus 13.1%), thus confirming the strong impact of this practice.

Veal meat was shown to be the least contributor (among different meat types) to the exposure of humans to ESC-R *E. coli* (21), and bovine meat was considered a minor source (3.6%) of human intestinal carriage of ESC-R *E. coli* compared to human-to-human contacts (60.1%) (22). Nevertheless, maintaining efforts in this sector to lower the global burden of resistance remains mandatory. In France, the implementation since 2012 of national action plans (NAPs) (called EcoAntibio) to tackle the AMR problem led to a 48% decrease in the global exposure of animals to antibiotics, and a >90% decrease in the exposure to last-generation cephalosporins (23). Improvements in antibiotic use practices in the veal production sector (including birth farms) have led to a decrease in antibiotic resistance over the past decade. As shown by the Resapath network, the proportion of clinical ESC-R *E. coli* in diseased veal calves decreased from 8% to 3% between 2013 and 2023 (5). However, our results showed that proportions of ESC-R *E. coli* collected from healthy calves in birth farms (12.5%), at their entry into the sorting center (17.5%), and when they leave the center (15.9%) to be sent to fattening farms, are still high. This healthy carriage provides fertile ground for AMR dissemination in fattening farms, after amplification by the use of antibiotics when calves are grouped. Consequently, lowering this proportion of resistant isolates is important, since mathematical models showed that minimizing AMR prevalence upon arrival at the fattening farm was an important factor to mitigate ESC-R carriage during the fattening process (24).

Twenty veal calves were ESC-R carriers at J1, which belonged to a low number of different bacterial clones (*n* = 5), with the overrepresentation of ST8095 (*n* = 10) and ST117 (*n* = 6). Of the 20 calves positive at J1, 17 remained positive at J2 and J3, and in 15 cases with the same clone. Moreover, both clones also had the capacity to persist over time, since identical bacteria were found at a 4- or 6-month interval in two farms. The colonization by the same bacterial clone of newborn calves not only within a farm but also from farms located in the same geographic area suggests inter-farm spread of AMR. There are many hypotheses to explain this spread, including *via* veterinarians, inseminators, mutual aid between farmers, equipment loan, or other causes that have not yet been identified. This spread appears to be significant, since approximately one in two farms (51.6%, 16/31) was positive. In addition, animal transport is likely to contribute to AMR dissemination, even though to a lesser extent, since ten negative calves on J1 (6.3%, 10/160) became positive after travel on J2 (of which five remained positive on J3). Nevertheless, we cannot exclude in this case that animals’ fecal samples only presented a low number of resistant bacteria at J1 and that the ESBL/AmpC isolates thus went undetected. Interestingly, the proportion of ESC-R-positive calves among animals sharing the same pen (A1–A4; 15.9%) as the index calves was comparable to that of from the index calves (J3; 16.3%) when they left the sorting center. However, the diversity of clones identified was much higher (*n* = 35, with the occurrence of 16 ST88 isolates) among A1–A4 calves, even though ST117 (*n* = 6) and ST8095 (*n* = 9) were still present. This might indicate geographical specificities rather than the dissemination of a few clones across the whole calf sector.

Over the 176 ESC-R *E. coli*, 97 presented an ESBL phenotype (55.1%) while 79 presented an AmpC phenotype (44.9%), and no isolate presented both phenotypes. This was surprising since most of the studies published until now on ESC-R *E. coli* in calves, including in France, reported mostly (if not exclusively) ESBL phenotypes. Among clones presenting an AmpC phenotype, the ST8095 reported here is rare in the literature. However, it is a single-locus variant (SLV) of the ST362 clone, which has recently been identified as an AmpC producer in 16/20 calves and a German farm (12). The second most frequent AmpC producer identified here was ST88, which has already been shown to be globally distributed in both humans and animals, including calves (9, 11, 16, 25, 26), associated with either *ampC* overexpression or the presence of *bla*_CTX-M_ genes. This shift from ESBL to AmpC phenotypes might be due to the modification in the use of veterinary antibiotics over the NAP. Among ESBL-producing *E. coli*, the *bla*_CTX-M-1_ gene was by far the most frequent one (*n* = 54, 55.7%), while the *bla*_CTX-M-14_ was less frequent (11.3%). This is partially due to the overrepresentation of the *bla*_CTX-M-1_/ST117 clones (*n* = 20). This ST117 clone, first strongly associated with the avian host (27), has nowadays disseminated globally and has been recurrently identified in calves (9, 16).

One limitation of this study is that the only indicator of resistance used here was the presence of ESC-R *E. coli*, which is an internationally recognized marker for such studies. However, in France, animal exposure to these antibiotics has been reduced by more than 90% in all species (23), thereby significantly reducing the occurrence of ESC-R isolates. Conversely, Resapath data (https://data-services.anses.fr/resapathfr/) are still showing a very high prevalence of resistance to first-generation antibiotics in calves (e.g., 80% resistance to amoxicillin). It can therefore be assumed that the indicator used here (i.e., ESC-R *E. coli*) only showed the lowest level of AMR burden in the calf sector. Studies using other markers, such as a combination of non-critical antibiotics to define multi-drug resistance (28), or by characterizing the presence of genes at the microbiome level, could be useful in addition to current practices.

Since certain ESC-R clones were largely distributed among different farms (suggesting between-farm clonal transmission), and since ESC-R *E. coli* were identified in numerous different farms, we assume that implementing good hygiene procedures on farms should be considered as a key factor for mitigating AMR in the veal sector. It most likely relates to common practices in dairy farms, with numerous actors often getting in and out of farms without strict biosecurity measures. Contrary to what is commonly believed in the veal sector, our results do not support the idea that a limited number of farms would be responsible for the overall situation due to unfavorable practices (heavy antibiotic use, use of milk containing antibiotic residues) by systematically providing calves with a poor health status to the fattening farms. The responsibility rather seems to be shared by a large number of farms (including organic ones), relatively independently of their individual practices. This situation highlights to what extent biosecurity levels strongly differ among animal production sectors, such as in industrial ones (broilers or laying hens, for instance) and more open systems (dairy cattle, for instance), with a direct impact on AMR spread.

### Conclusion

This study showed that 12.5% of the veal calves were ESC-R carriers in their farm of origin, while 6.2% of the additional animals became carriers during their transport to the sorting center. In this study, a few specific clones (ST8095, ST117) were responsible for the AMR dissemination across farms, and a surprisingly important proportion of AmpC producers contributed to the overall ESC-R resistance. Altogether, 15.9% of the calves carried an ESC-R *E. coli* isolate at the departure from the sorting center. It is thus clear that AMR rates in fattening farms result from cumulative effects of all risk factors along the whole production chain. It includes feeding calves with antibiotic residue-containing waste milk, animal grouping, and, as demonstrated here, a significant lack of biosecurity measures in dairy farms.

## Data Availability

The project was deposited in GenBank under BioProject accession number PRJNA1367335.

## SUPPLEMENTAL MATERIAL

Table S1 spectrum.00659-26-s0001.xlsx Description of the whole sampling.

Table S2 spectrum.00659-26-s0002.xlsx Farm questionnaire.

Table S3 spectrum.00659-26-s0003.xlsx Quality controls.

Table S4 spectrum.00659-26-s0004.xlsx Distance matrix for main STs.

Table S5 spectrum.00659-26-s0005.xlsx NGS data.

Table S6 spectrum.00659-26-s0006.docx List of all STs identified.

ASM does not own the copyrights to Supplemental Material that may be linked to, or accessed through, an article. The authors have granted ASM a non-exclusive, world-wide license to publish the Supplemental Material files. Please contact the corresponding author directly for reuse.
