# Supplementary material for: Extended-spectrum beta-lactamases from French veal calves: WGS-based data will help build targeted AMR mitigation strategies
Source: Microbiol Spectr. 2026 Jun 22;14(8):e00659-26. doi: 10.1128/spectrum.00659-26 (PMC13436377; doi:10.1128/spectrum.00659-26)
Supplement: Supplementary file 1 [file spectrum.00659-26-s0006.docx]

Table S6. Genetic diversity among ESBL- and AmpC-producing *E. coli* in veal calves

| ST | No. of ESBL-Ec | % ESBL-Ec | No. of AmpC-Ec | % AmpC-Ec | Total no. of isolates | % total no. of isolates |
| --- | --- | --- | --- | --- | --- | --- |
| 10 | 8 | 8.2 | 0 | 0.0 | 8 | 4.5 |
| 17 | 1 | 1.0 | 0 | 0.0 | 1 | 0.6 |
| 23 | 2 | 2.1 | 2 | 2.5 | 4 | 2.3 |
| 46 | 10 | 10.3 | 0 | 0.0 | 10 | 5.7 |
| 48 | 2 | 2.1 | 0 | 0.0 | 2 | 1.1 |
| 56 | 0 | 0.0 | 1 | 1.3 | 1 | 0.6 |
| 57 | 3 | 3.1 | 0 | 0.0 | 3 | 1.7 |
| 58 | 3 | 3.1 | 6 | 7.6 | 9 | 5.1 |
| 69 | 6 | 6.2 | 0 | 0.0 | 6 | 3.4 |
| 69* | 1 | 1.0 | 0 | 0.0 | 1 | 0.6 |
| 85 | 0 | 0.0 | 1 | 1.3 | 1 | 0.6 |
| 88 | 1 | 1.0 | 18 | 22.8 | 19 | 10.8 |
| 117 | 24 | 24.7 | 0 | 0.0 | 24 | 13.6 |
| 154 | 1 | 1.0 | 0 | 0.0 | 1 | 0.6 |
| 164 | 1 | 1.0 | 0 | 0.0 | 1 | 0.6 |
| 167 | 2 | 2.1 | 0 | 0.0 | 2 | 1.1 |
| 301 | 8 | 8.2 | 3 | 3.8 | 11 | 6.3 |
| 349 | 0 | 0.0 | 1 | 1.3 | 1 | 0.6 |
| 362 | 1 | 1.0 | 0 | 0.0 | 1 | 0.6 |
| 540 | 1 | 1.0 | 0 | 0.0 | 1 | 0.6 |
| 744 | 1 | 1.0 | 0 | 0.0 | 1 | 0.6 |
| 765 | 1 | 1.0 | 0 | 0.0 | 1 | 0.6 |
| 783 | 0 | 0.0 | 8 | 10.1 | 8 | 4.5 |
| 925 | 0 | 0.0 | 2 | 2.5 | 2 | 1.1 |
| 973 | 1 | 1.0 | 0 | 0.0 | 1 | 0.6 |
| 1152 | 1 | 1.0 | 0 | 0.0 | 1 | 0.6 |
| 1266 | 3 | 3.1 | 0 | 0.0 | 3 | 1.7 |
| 1433 | 1 | 1.0 | 0 | 0.0 | 1 | 0.6 |
| 1722 | 1 | 1.0 | 0 | 0.0 | 1 | 0.6 |
| 3018 | 1 | 1.0 | 0 | 0.0 | 1 | 0.6 |
| 3714* | 1 | 1.0 | 0 | 0.0 | 1 | 0.6 |
| 3995 | 0 | 0.0 | 1 | 1.3 | 1 | 0.6 |
| 4981 | 7 | 7.2 | 0 | 0.0 | 7 | 4.0 |
| 5926 | 2 | 2.1 | 0 | 0.0 | 2 | 1.1 |
| 8095 | 0 | 0.0 | 36 | 45.6 | 36 | 20.5 |
| 8622 | 1 | 1.0 | 0 | 0.0 | 1 | 0.6 |
| ND | 1 | 1.0 | 0 | 0.0 | 1 | 0.6 |
| total | 97 |  | 79 |  | 176 |  |

*single-locus variant of the stated ST
